# ODIASP: An Open‐Source Software for Automated SMI Determination—Application to an Inpatient Population

**DOI:** 10.1002/jcsm.70023

**Published:** 2025-07-26

**Authors:** Katia Charrière, Antoine Ragusa, Béatrice Genoux, Antoine Vilotitch, Svetlana Artemova, Charlène Dumont, Paul‐Antoine Beaudoin, Pierre‐Ephrem Madiot, Gilbert R. Ferretti, Ivan Bricault, Eric Fontaine, Jean‐Luc Bosson, Alexandre Moreau‐Gaudry, Joris Giai, Cécile Bétry

**Affiliations:** ^1^ Public Health Department Univ. Grenoble Alpes, Clinical Investigation Center‐Technological Innovation, INSERM CIC1406, CHU Grenoble Alpes Grenoble France; ^2^ Univ. Grenoble Alpes, Méthodologie de l'information en Santé, Biostatistiques, Recherche clinique et Innovation Technologique, Pôle Santé Publique, CHU Grenoble Alpes Grenoble France; ^3^ Univ. Grenoble Alpes, CNRS, UMR 5525, VetAgro Sup, Grenoble INP, TIMC Grenoble France; ^4^ Univ. Grenoble Alpes, Méthodologie de l'information en Santé, Biostatistiques, Recherche clinique et Innovation Technologique, Pôle Santé Publique, Unité d'ingénierie des données, Pôle Santé Publique, Grenoble Alpes University Hospital Grenoble France; ^5^ Univ. Grenoble Alpes, Direction des Services Numériques, Grenoble Alpes University Hospital Grenoble France; ^6^ Univ. Grenoble Alpes, INSERM U1209, IAB, CHU Grenoble Alpes, Service de radiologie diagnostique et interventionnelle Grenoble France; ^7^ Department of Endocrinology, Diabetology and Nutrition Univ. Grenoble Alpes, INSERM U1055, LBFA, CHU Grenoble Alpes Grenoble France; ^8^ Department of Endocrinology, Diabetology and Nutrition Univ. Grenoble Alpes, CNRS, UMR 5525, VetAgro Sup, Grenoble INP, CHU Grenoble Alpes, TIMC Grenoble France

**Keywords:** body composition, computational neural networks, computer‐assisted, image processing, malnutrition, sarcopenia, skeletal muscle

## Abstract

**Background:**

The diagnosis of malnutrition has evolved with the GLIM recommendations, which advocate for integrating phenotypic criteria, including muscle mass measurement. The GLIM framework specifically suggests using skeletal muscle index (SMI) assessed via CT scan at the third lumbar level (L3) as a first‐line approach. However, manual segmentation of muscle from CT images is often time‐consuming and infrequently performed in clinical practice. This study is aimed at developing and validating an open‐access, simple software tool called ODIASP for automated SMI determination.

**Methods:**

Data were retrospectively collected from a clinical data warehouse at Grenoble Alpes University Hospital, including epidemiological and imaging data from CT scans. All consecutive adult patients admitted in 2018 to our tertiary centre who underwent at least one CT scan capturing images at the L3 vertebral level and had a recorded height were included. ODIASP combines two algorithms to automate L3 slice selection and skeletal muscle segmentation, ensuring a seamless process. Agreement between cross‐sectional muscle area (CSMA) values obtained using ODIASP and the reference methodology (i.e., manual determination) was evaluated using the intraclass correlation coefficient (ICC). The prevalence of reduced SMI was also assessed.

**Results:**

SMI was available for 2503 participants, 53.3% male, with a median age of 66 years (51–78) and a median BMI of 24.8 kg/m^2^ (21.7–28.7). In a validation subset of 674 scans, agreement between the reference method and ODIASP was substantial (ICC: 0.971; 95% CI: 0.825–0.989) and improved to excellent (ICC: 0.984; 95% CI: 0.982–0.986) after correcting for systematic overestimation (a 5.8 cm^2^ [5.4–6.3]) indicating excellent agreement. The prevalence of reduced SMI was 9.1% overall (11.0% in men and 6.6% in women). The ODIASP software is available as a downloadable executable to support its use in research settings.

**Conclusions:**

This study demonstrates that ODIASP is a reliable tool for automated SMI at the L3 vertebra level from CT scans. The integration of validated AI algorithms into a simple, open‐source software enables scalable, standardised assessment of SMI in diverse patient populations and supports future integration into clinical workflows for improved nutritional assessment.

## 1 Introduction

Since the release of the GLIM recommendations, malnutrition diagnosis has been based on both phenotypic and etiological criteria [1]. In current clinical practice, phenotypic criteria for malnutrition primarily involve body mass index (BMI) and weight loss assessments [2, 3]. However, a large proportion of patients are not weighed during hospitalization [4]. The GLIM framework recommends that muscle mass assessments should be performed as a first‐line approach to detect muscle mass reduction using one of three methods: dual‐energy x‐ray absorptiometry (DEXA), computerised tomography (CT) scan or bioelectrical impedance analysis (BIA). BIA provides fat‐free mass, an indirect estimate of muscle mass, that is highly sensitive to hydration status. Moreover, BIA‐derived values rely on device‐ and population‐specific equations, limiting their generalizability in diverse clinical settings. DEXA is considered more reliable but remains poorly available for routine body composition assessment in clinical practice due to cost and accessibility constraints. In contrast, CT is routinely performed in hospital settings and is thus promising for opportunistic malnutrition screening. A growing body of literature supports the use of CT to assess muscle mass reduction by measuring the cross‐sectional muscle area (CSMA) at the third lumbar (L3) vertebra, from which the skeletal muscle index (SMI) is derived. However, the widespread use of CT for this purpose faces two major barriers: first, the manual determination of CSMA is time‐consuming and requires trained personnel; second, there is a lack of validated SMI cut‐off values for determining reduced muscle mass [3]. Developing an automated solution for CSMA determination is essential to overcome both barriers, enabling large‐scale, standardised assessments and facilitating the development of reference values across clinical populations.

AI algorithms have the potential to automate SMI determination, potentially extending the use of CT scan for malnutrition diagnosis in clinical practice and across large volumes of CT scans [5]. Although research in AI‐driven CT scan analysis is expanding, a significant gap persists between technological advancements and clinical integration. We hypothesise that part of this gap arises from the lack of user‐friendly interfaces that facilitate the application of these algorithms. Furthermore, few comprehensive solutions exist that provide a fully automated pipeline for SMI determination, including both L3 slice identification and muscle segmentation. Existing tools are often developed within specific cohorts, limiting their applicability for malnutrition screening in broader, unselected patient populations [6, 7, 8].

This study is a part of a larger research project: the Optimization of the DIAgnosis of SarcoPenia (ODIASP) through the automated determination of SMI study. The primary objective of the present study was to develop and validate a simple, open‐access software for the automated determination of the SMI in clinical research. Additionally, we aimed to evaluate the prevalence of reduced SMI within a cohort of patients managed at a tertiary hospital.

## 2 Methods

### 2.1 Ethics Statement

The ODIASP study was conducted in accordance with the ethical standards laid down in the 1964 Declaration of Helsinki and its later amendments. Ethical approval was obtained on 18 August 2021 by the regional ethics committee (CECIC Rhône‐Alpes‐Auvergne, Clermont‐Ferrand, IRB 5891). In compliance with current French legislation for retrospective studies using clinical data, participants were individually informed that their data could be used for research purposes (in line with the MR‐004 CNIL reference methodology). Individuals who objected to the use of their data or those with incomplete or outdated contact information were excluded from the study.

### 2.2 Study Participants

All consecutive participants aged 18 years and older, admitted to Grenoble University Hospital (CHU Grenoble Alpes) between January and December 2018, who underwent at least one CT scan potentially capturing images at the L3 vertebral level and who had a recorded height were retrospectively included in the study. Participants with nonabdominal CT scans, including spinal or thoracic CT scans, were also eligible, as these may contain images at the L3 level. This inclusion was particularly relevant for assessing whether the ODIASP software could effectively detect CT scans that lacked an L3 slice. When multiple CT scans were available for a patient during the inclusion period, the scan most likely to include the L3 slice was selected for analysis. A subset of this population, previously defined for preliminary research [9], was used for validation purposes and is hereafter referred to as the validation subset.

### 2.3 Data Collection

Data were retrospectively collected from the clinical data warehouse (CDW) PREDIMED (French acronym for *Plateforme de Recueil et d'Exploitation des Données bIoMEDicales*), implemented at the Grenoble Alpes University Hospital [10, 11]. All structured data were pseudonymized, and CT scans were deidentified. This deidentification of CT scans was carried out by (1) removing all identifiable Digital Imaging and Communication in Medicine (DICOM) tags following the basic profile recommendations of the DICOM standard^1^ and (2) excluding all derived images (i.e., images where pixel values are derived or computed from other images, such as screenshots or dose reports) based on the ‘ImageType’ DICOM attribute.

The following data were collected:

- General data: age, sex, height and weight.
- Imaging data: CT scans, including the abdominal area, in DICOM format with metadata. Since contrast enhancement has a minor impact on CSMA, both CT images with and without contrast administration were eligible [12, 13, 14]. CT images could originate from different CT machines (Optima CT660 GE Healthcare, Revolution CT GE Healthcare, Siemens Somatom Definition Edge, Revolution HD GE Healthcare, Revolution EVO GE Healthcare, Toshiba Aquilion and Siemens Somatom Definition AS+).

### 2.4 CSMA and SMI Determination

#### 2.4.1 Reference Method

All processing was performed on a single GPU machine (NVIDIA TITAN RTX 16 graphics card, a 3.7 GHz CPU and 64 GB of RAM) with an Intel(R) Xeon(R) W‐2135 processor. The L3 vertebra was manually identified by a medical expert on the sagittal reconstruction using the Picture Archiving and Communication System (PACS) in DICOM format. For each participant, a slice approximately halfway along the vertebra was selected for muscle segmentation. The upper and lower extremities of the vertebra were also identified to later validate whether the slice selected by the ODIASP tool was indeed located at the L3 level. Skeletal muscle was then manually segmented with SliceOmatic Version 5.0 (Tomovision, Canada) according to previously published methods to obtain the CSMA [9]. The abdominal muscles (transversus abdominis, external and internal obliques and rectus abdominis), the paraspinal muscles (erector spinae and quadratus lumborum) and the psoas muscle were segmented using Hounsfield unit (HU) values ranging from −29 to 150 [15].

#### 2.4.2 ODIASP Tool

**FIGURE 1:**
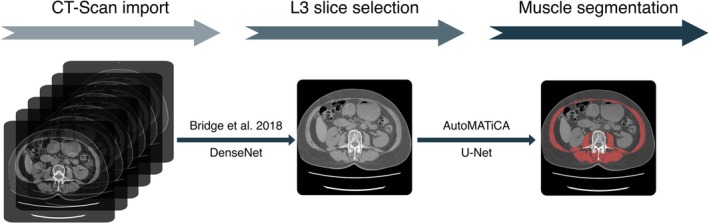
Schematic overview of the architecture of the ODIASP tool. The ODIASP tool is based on two previously published algorithms from Bridge et al. [16, 17] and Paris et al.

The ODIASP tool integrates two open‐source algorithms: (1) for the automatic selection of a slice at the level of the L3 vertebra and (2) for the automatic segmentation of skeletal muscle in this slice (Figure 1). For the first step, we employed the L3 slice selection algorithm developed by Bridge et al. [16, 17]. The algorithm was developed using five‐fold cross‐validation, following the ‘*K*‐fold, folded test set’ approach, on the validation subset of 676 manually annotated CT scans. For training, scan volumes spanning up to 400 mm cranially and 250 mm caudally from the L3 level were used. The DenseNet model was trained to predict the distance to the L3 slice defined by the clinician using the methodology and source code provided by Bridge et al. Hyperparameters were optimised on the first fold but showed minimal impact on model performance and were therefore kept unchanged for the remaining folds. The final configuration included a batch size of 64 images, a learning rate of 0.001 and no dropout (rate of 0), while other parameters followed those proposed by Bridge et al. Each training run was conducted over 80 epochs. This cross‐validation strategy allowed us to evaluate model performance across the entire dataset and to verify the consistency of the results. For the second step, we utilised the AutoMATiCA algorithm developed by Paris et al. [18].

### 2.5 SMI Determination

The test and validation datasets were processed using ODIASP, without any subsequent manual validation of the CSMA. The only excluded cases, referred to as ‘ODIASP failures’, were those in which the software did not detect a slice at the L3 level. The SMI was calculated as the CSMA (in square centimetres) divided by height squared (in square metres).

### 2.6 Statistical Analysis

Characteristics of the cohort were presented as medians with interquartile ranges (IQRs) or using frequencies and proportions depending on their nature. Both steps of the ODIASP tool were individually validated prior to evaluating the full pipeline. All validation procedures were conducted on the validation subset. For the first step, the trained algorithm by Bridge et al. was evaluated by calculating the percentage of slices correctly located between the lower and upper extremities of the L3 vertebra, as well as the number of exactly matching slices and the median distance between the automatically and manually selected L3 slices. For the second step, AutoMATiCA was previously validated on ODIASP project CT data using the Dice similarity coefficient (DSC) to assess the overlap between automated and manual segmentations [9]. Finally, for the validation of the complete ODIASP pipeline, agreement between the CSMA values obtained with ODIASP and those from the reference method (i.e., manual segmentation) was assessed using the intraclass correlation coefficient (ICC) along with 95% confidence intervals. To account for systematic errors, the fixed bias was removed so that only the random residual errors were retained for computing the ICC. The systematic error is illustrated in a Bland–Altman plot. This analysis was complemented with a mixed regression model. The interpretation of the strength of agreement between the two methods was based on the criteria established by Koo and Li: an ICC value < 0.5 indicated poor reliability, a value between 0.5 and 0.75 indicated moderate reliability, a value between 0.75 and 0.90 indicated substantial reliability and a value > 0.90 indicated excellent reliability [19]. For determining the reduction in SMI within our sample, thresholds were derived from the study by van der Werf et al. [20] and are applicable only to the subpopulation aged 20–79 years with a BMI ranging from 17 to 35 kg/m^2^. Statistical analyses were conducted using Stata (Version 15) with *p* values < 0.05 considered statistically significant and without adjustment for test multiplicity.

## 3 Results

### 3.1 Participant Inclusion and Characteristics

**FIGURE 2:**
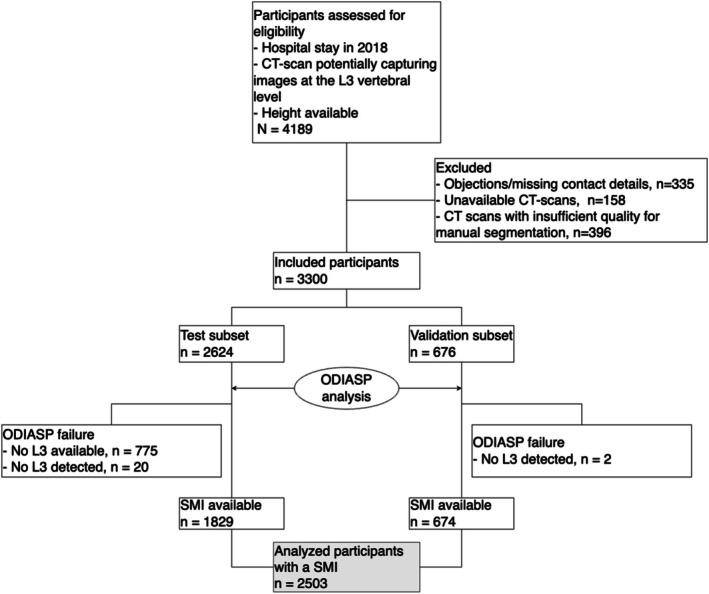
Flow chart of study participants.

A total of 3300 participants were included, and 2503 participants were analysed (Figure 2). Their median age was 66 (51–78) years, with 1334 (53.3%) males. The median BMI was 24.8 kg/m^2^ (21.7–28.7), with 174 (7.3%) having a BMI < 18.5 kg/m^2^ and 459 (19.3%) classified as obese (BMI ≥ 30 kg/m^2^). The median SMI was 45.1 cm^2^/m^2^ (38.4–52.5). Participants were hospitalised in the following units: day hospital (11.0%), medicine (27.4%), geriatrics (2.8%), surgery (10.4%), intensive care (6.1%), rehabilitation (1.8%) and emergency care (40.5%).

### 3.2 Validation of the ODIASP Tool

The ODIASP tool operates through two main steps: first, identifying a L3 slice, and second, segmenting the skeletal muscle (Figure 1). For the first step, we validated the algorithm from Bridge et al. The algorithm correctly identified the L3 slice in 88% of cases (95% CI: 85.49–90.50, *n* = 596), and the exact same L3 slice was selected by both the algorithm and the expert in 67 cases (9.9%). Across the entire validation subset, the median distance between the automatically and manually selected slices was 0 mm (IQR: −3.8 to 5.0). For slices outside the L3 level (*n* = 80), the median distance from the chosen L3 slice was −5.6 mm (−17.5 to 12.5), indicating that the slices were, on average, 5.6 mm below the lower extremity of the L3 vertebra. The algorithm for the second step, muscle segmentation, was previously validated externally [9].

**FIGURE 3:**
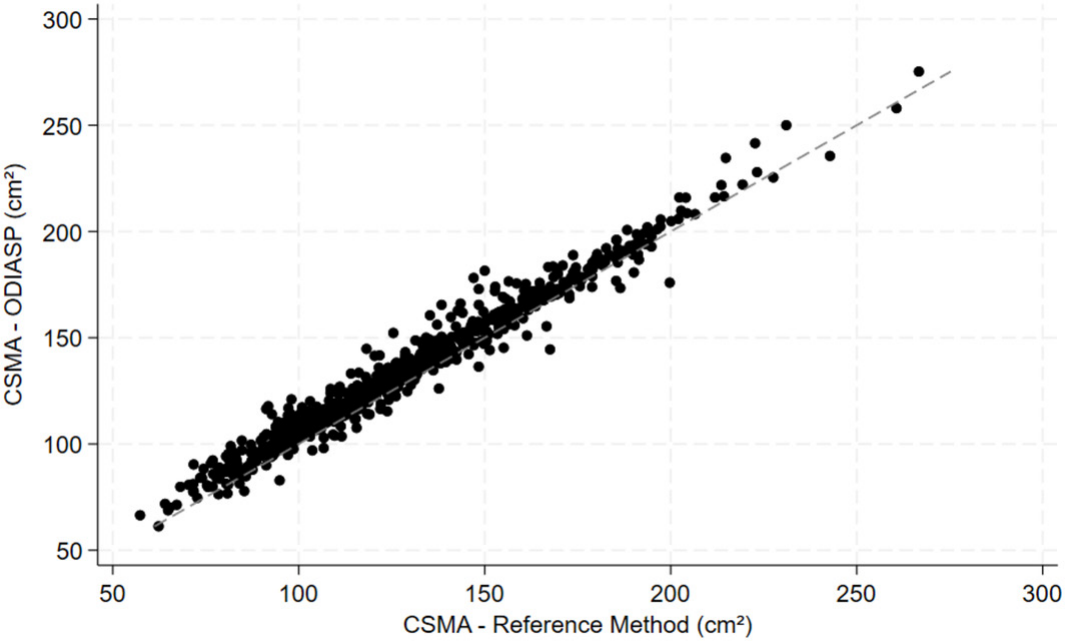
Scatterplot comparing cross‐sectional muscle area (CSMA) measurements obtained with the reference method and the ODIASP tool (*n* = 674). Each data point represents an individual measurement, and the dashed line signifies perfect agreement between the two methods. The proximity of data points to the dashed line indicates a high degree of agreement.

To validate the entire pipeline, we compared the CSMA obtained using the reference method with that generated by the ODIASP tool in the validation subset. ODIASP failed to provide results for two participants (Figure 2). There was substantial agreement between the reference method and ODIASP (ICC: 0.971; 95% CI: 0.825–0.989) (Figure 3). After correcting for systematic errors, that is, by retaining only the random residual error for the ICC (a 5.8 cm^2^ [5.4–6.3] overestimation of the CSMA, as illustrated in the Bland–Altman—Figure S1), there was excellent agreement (ICC: 0.984, 95% CI: 0.982–0.986). This corrected agreement remained excellent across subgroups, with ICC values stratified by sex, BMI and contrast enhancement (Table S1).

### 3.3 An Open‐Access and Simple Interface

**FIGURE 4:**
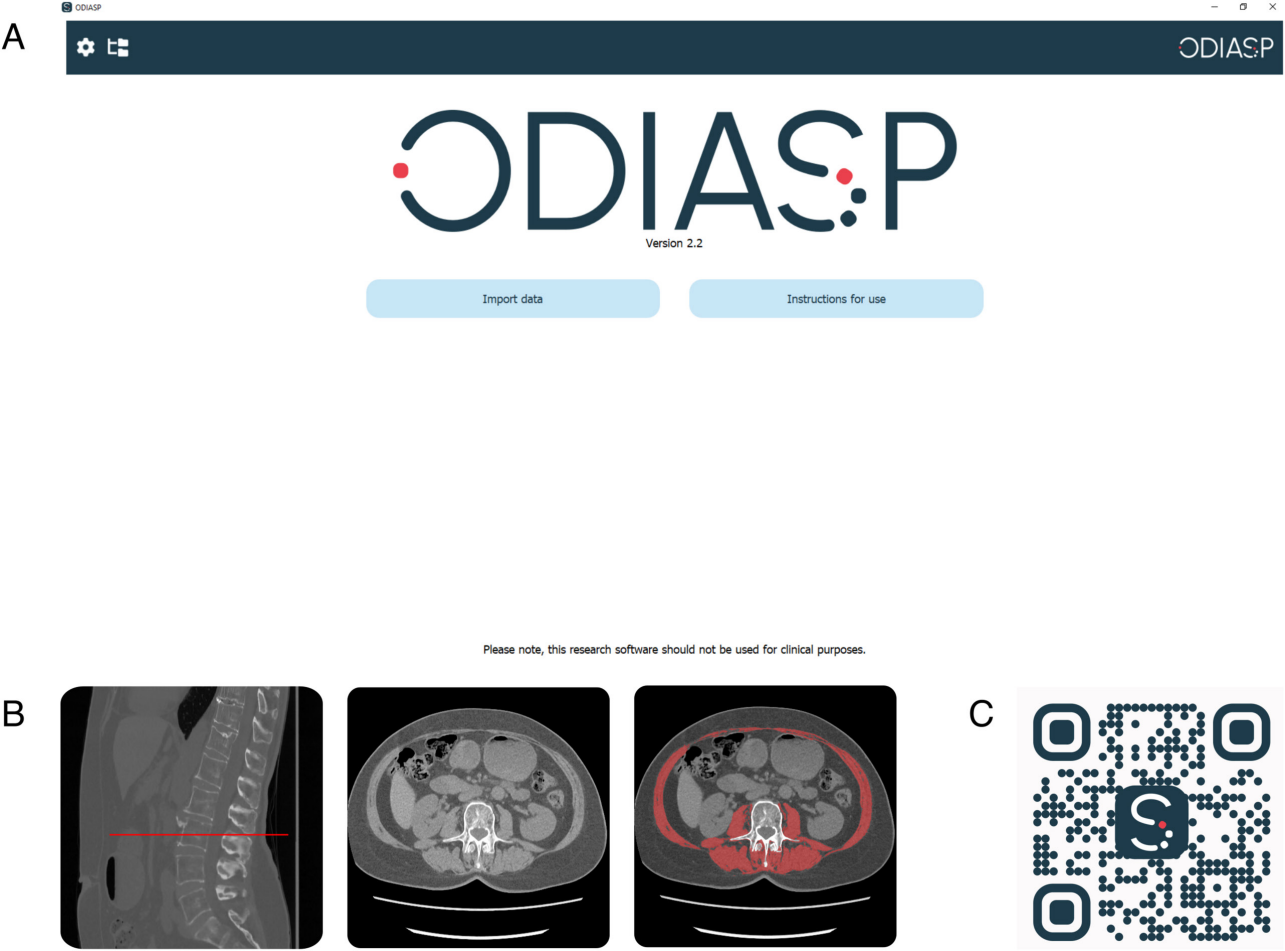
The ODIASP tool: an open access software with user‐friendly interface. (A) User interface. (B) Example of muscle segmentation. (C) QR code for downloading the ODIASP software.

To facilitate the use of the ODIASP tool, we encapsulated the code into a simple software interface (Figure 4A). The interface displays the sagittal view with the chosen L3 slice localisation, the L3 slice itself and its segmentation, allowing for easy visual validation of results (Figure 4B). This software is freely available as a downloadable executable at https://odiasp.timc.fr/ (Figure 4C).

ODIASP allows processing of large sets of CT scans without the need for preprocessing. It takes approximately 4 min to analyse one CT scan. In some cases, ODIASP may not correctly identify or segment muscle (see examples of random segmentation in Figure S2). However, the interface allows for quick visual inspection of the results if necessary.

### 3.4 Prevalence of Reduced SMI in a Large Cohort of Hospitalised Participants

Based on previously published cut‐off values for individuals aged 20–79 years with a BMI between 17 and 35 kg/m^2^, 103 of 937 men (11.0%) and 46 of 699 women (6.6%) were considered to have reduced skeletal muscle mass, representing a total of 9.1% of the participants with an available cut‐off [20].

## 4 Discussion

This study was aimed at developing and validating a tool for the automated determination of SMI at the L3 vertebra level using a CT scan in a large population. We integrated AI algorithms into a simple software, making advanced technologies accessible to clinical researchers who may be unfamiliar with them.

### 4.1 Reliability of the ODIASP Automated Tool

Most previous studies have focused on tissue segmentation at the L3 level [18, 21, 22] or for 3D muscle segmentation [23], with few addressing the challenge of developing a fully automated pipeline for L3 SMI determination, including the identification of the appropriate L3 slice. To our knowledge, we are the first to develop and validate automated AI software for L3 SMI determination in an unselected population of patients managed at a tertiary hospital across various units, including emergency and intensive care. Previous tools have been developed for specific populations, such as cancer patients [6, 7] or mixed cohorts including cancer, sepsis and healthy subjects [8]. Our approach involved utilising previously published algorithms and validating them in our cohort, demonstrating good reliability across a large sample. We achieved accurate identification of most L3 slices, with 88% correctly positioned at the L3 vertebra, which aligns with previous findings (Delrieu et al.: 91.2% and 74.1% in, two datasets [6]). Slices identified outside of the L3 level showed a median deviation of −5.6 mm (−17.5; 12.5), typically locating them at adjacent vertebrae, given the approximate height of vertebrae (30 mm) and intervertebral disks (10 mm) [24]. A prior study indicates that CSMA at neighbouring vertebrae is relatively comparable [25]. The reliability of muscle segmentation was previously validated [9], and our full pipeline demonstrated substantial to excellent reliability.

### 4.2 Clinical Implications

The prevalence of reduced SMI has mainly been studied in cancer populations [26, 27, 28]. To our knowledge, our study is the first to assess it in a large cohort of unselected patients, with the sole inclusion criteria being the availability of a CT scan. The cut‐offs for defining reduced SMI remain controversial, while the cut‐offs proposed by Prado et al. [15] have been widely used; they are specific to patients with obesity and cancer and can lead to an overestimation of reduced SMI prevalence [29]. In our study, we referred to the cut‐offs by van der Werf et al. [20], which are applicable to a Caucasian population and align well with our predominantly Caucasian cohort. Furthermore, van der Werf et al. provide age‐ and BMI‐specific cut‐offs, enhancing the relevance of our findings. Our research indicates that 9% of the patients in our tertiary hospital setting have reduced muscle mass. However, since the selected cut‐off values have not yet been validated against clinical outcomes, as currently recommended, the estimated prevalence should be considered a first approximation [3]. Additionally, our tool tends to slightly overestimate CSMA by a few square centimetre. We hypothesise that this slight overestimation is partly explained by the inclusion of adjacent nonmuscular structures, such as bowel loops or vascular elements, that share similar HU densities and are occasionally segmented by default by the AutoMATiCA algorithm integrated in ODIASP. While the difference of SMI is minimal once normalised for height [2], we cannot exclude the possibility that some patients close to the threshold were misclassified as having normal muscle mass. Moreover, as no manual review was performed, potential segmentation inaccuracies may also have contributed to misclassification.

### 4.3 Study Limitations

Several limitations should be acknowledged. First, we trained the algorithm of Bridge et al. on our dataset since it was only available in an untrained version. Consequently, our tool lacks full external validation. This internal validation may lead to an overestimation of model performance due to potential data‐specific biases. Therefore, further testing on independent external datasets is required to assess the generalisability and robustness of the tool in other clinical environments. Second, when testing ODIASP on the analysed populations, we identified some processing failures related to the lack of L3 slice determination. These issues could be due to insufficient CT scan quality or limitations of the ODIASP tool itself. Poor‐quality scans, such as those with photon starvation or anatomical anomalies, may affect segmentation accuracy. While ODIASP could be further improved to better handle such challenging cases, we believe that human oversight remains essential for maintaining data quality; thus, our software provides features for visual manual validation, in line with the recent European Union AI Act, which emphasises the necessity of human oversight in algorithmic decision‐making [30]. Third, processing large datasets can be time‐consuming; for instance, analysing 1000 scans requires just over 24 h with a standard desktop computer. However, the process is fully automated and runs in the background. Our approach involved testing ODIASP under real‐life conditions without preprocessing the data, which can lengthen the process but reduces the time needed for data preparation. Additionally, it is important to acknowledge that some CT scans may have insufficient quality for accurate segmentation, a factor that should be considered in future clinical research. Lastly, ODIASP tends to slightly overestimate the CSMA compared to the ground truth. This discrepancy reflects a systematic error comparable to interexpert variability and could be partly influenced by the overestimation observed with AutoMATiCA or the fact that the ground truth was constructed at our centre by only two experts [9, 18].

### 4.4 Future Directions

Future work will focus on enhancing the reliability of our software. A strength of this project is our access to diverse medical imaging data through the CDW PREDIMED, allowing for the inclusion of patients from various units and with different pathologies. However, our study is monocentric, which may lead to overfitting and limit generalisability. To address this, we intend to establish collaborations with other centres that possess annotated CT scan datasets, with the aim of conducting external validation studies and assessing the reproducibility of our tool across diverse clinical settings. Although we anticipate good performance in external cohorts, potential discrepancies, if observed, could result from the use of traditional training methods, which are known to overestimate model accuracy due to hidden data acquisition bias‐induced shortcuts (DABIS). To strengthen the robustness of the tool, possible strategies include incorporating external datasets during training, as well as applying dedicated methods to mitigate biases even in the absence of external data [31]. Our objective is to develop extensive cohorts for studying malnutrition and sarcopenia in inpatients. However, many patients do not undergo abdominal CT scans. To address this limitation, we plan to enhance ODIASP's capabilities to analyse chest CT scans, considering the growing interest in assessing SMI at the T12 vertebra level [32]. We also plan to validate the segmentation of other tissues—intermuscular, visceral and subcutaneous adipose tissue—performed in AutoMATiCA before integrating it into ODIASP [18]. Furthermore, recent developments in deep learning now allow for 3D segmentation of body composition from CT scans using tools such as TotalSegmentator [23]. These approaches could enhance the precision of muscle mass evaluation compared to traditional 2D methods based on a single slice [33]. However, current diagnostic recommendations for malnutrition are based on the use of CSMA at the L3 level as a validated proxy for total muscle mass. In routine clinical settings, whole‐body CT scans remain uncommon, limiting the feasibility of such 3D methods for opportunistic screening. Further research is needed to define standardised anatomical locations and cut‐off values for partial‐body 3D segmentations and to assess whether these metrics outperform SMI in predicting clinical outcomes. Additionally, their clinical implementation will depend on the feasibility of real‐time processing, which remains a practical challenge for broader adoption. Finally, it is important to note that ODIASP is not yet CE marked or FDA approved, limiting its use to clinical research rather than routine practice. Our ultimate goal is to deploy this software in clinical settings after obtaining all mandatory regulatory authorisation. To this end, the software is currently being redeveloped under a quality assurance framework, in collaboration with a private partner specialised in regulatory support, with the aim of facilitating the CE marking process.

## 5 Conclusion

This study demonstrates that ODIASP is a reliable and fully automated tool for estimating SMI from CT scans by segmenting the CSMA at the L3 vertebra level. By integrating validated AI algorithms into a simple, open‐source software platform, ODIASP offers a scalable and standardised approach for assessing muscle mass in clinical research. The tool lays the foundation for large‐scale, harmonised evaluation of SMI, which is essential for establishing clinically validated cut‐off values. While the current version is limited to research use, it is being redeveloped under a quality assurance framework in preparation for regulatory approval, a key step toward clinical implementation. Ultimately, this work supports the integration of opportunistic screening for malnutrition and sarcopenia into routine clinical workflows, with the potential to enhance patient care through more accurate and accessible nutritional assessment.

## Ethics Statement

The ODIASP study was conducted in accordance with the ethical standards laid down in the 1964 Declaration of Helsinki and its later amendments. Ethical approval was obtained on 18 August 2021 by the regional ethics committee (CECIC Rhône‐Alpes‐Auvergne, Clermont‐Ferrand, IRB 5891). In compliance with current French legislation for retrospective studies using clinical data, participants were individually informed that their data could be used for research purposes (in line with the MR‐004 CNIL reference methodology).

## Conflicts of Interest

The authors declare no conflicts of interest.

## Supporting information


**Figure S1.** Bland–Altman plot comparing CSMA (square centimetres) measurements obtained by the reference method and the ODIASP tool. The solid line represents the mean difference (systematic bias), and the dashed lines indicate the 95% limits of agreement.
**Table S1.** Intraclass correlation coefficients (ICCs) between ODIASP and the reference method after correction for systematic error, stratified by sex, BMI categories and contrast enhancement.
**Figure S2.** Examples illustrating muscle segmentation at the L3 level on random CT scans processed by the ODIASP software.

## Endnotes

^1^

https://dicom.nema.org/medical/dicom/current/output/html/part15.html#sect_E.1.1
