# Supplementary material for: ODIASP: An Open‐Source Software for Automated SMI Determination—Application to an Inpatient Population
Source: J Cachexia Sarcopenia Muscle. 2025 Jul 26;16(4):e70023. doi: 10.1002/jcsm.70023 (PMC12677933; doi:10.1002/jcsm.70023)
Supplement: Supplementary file 1 — Figure S1. Bland–Altman plot comparing CSMA (square centimetres) measurements obtained by the reference method and the ODIASP tool. The solid line represents the mean difference (systematic bias), and the dashed lines indicate the 95% limits of agreement. Table S1. Intraclass correlation coefficients (ICCs) between ODIASP and the reference method after correction for systematic error, stratified by sex, BMI categories and contrast enhancement. Figure S2. Examples illustrating muscle segmentation at the L3 level on random CT scans processed by the ODIASP software. [file JCSM-16-e70023-s001.docx]

**
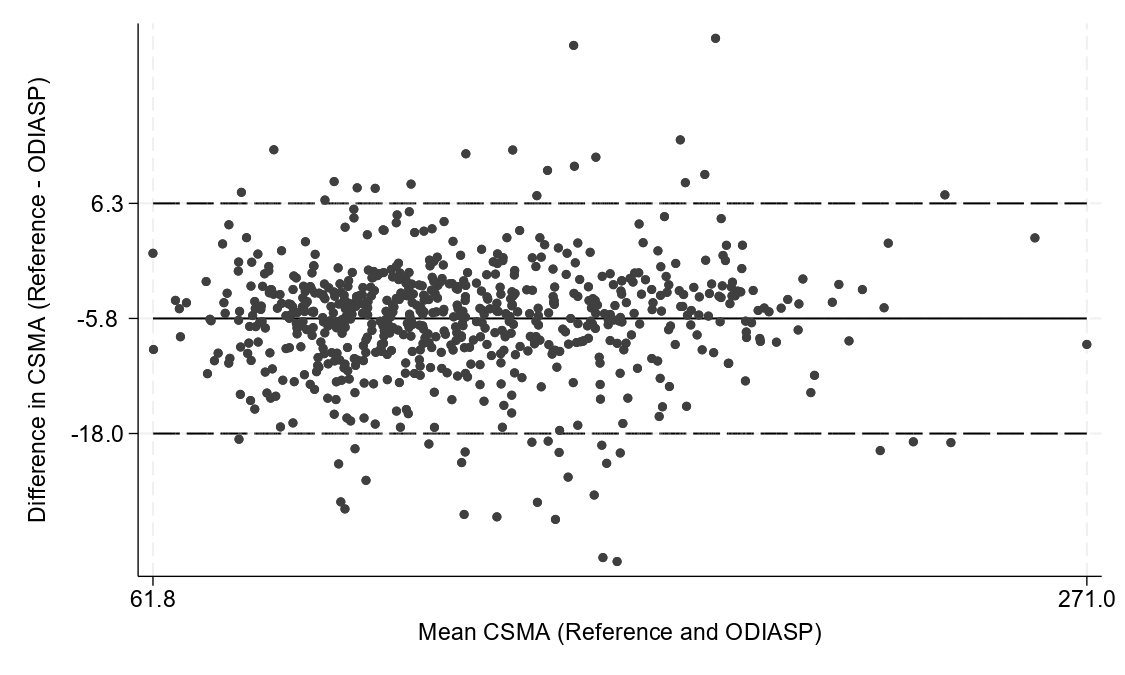
**

**Supplementary Figure 1. Bland-Altman plot comparing CSMA (**cm²) **measurements obtained by the reference method and the ODIASP tool.**

The solid line represents the mean difference (systematic bias), and the dashed lines indicate the 95% limits of agreement.

|  | ICC [95% CI] | N |
| --- | --- | --- |
| Males | 0.978 [0.974-0.982] | 362 |
| Females | 0.963 [0.954-0.970] | 312 |
| IMC ≥ 25 kg/m^2^ | 0.988 [0.985-0.990] | 306 |
| IMC < 25 kg/m^2^ | 0.974 [0.968-0.979] | 361 |
| With contrast enhancement | 0.982 [0.979-0.985] | 537 |
| Without contrast enhancement | 0.991 [0.988-0.994] | 137 |

Supplementary Table 1. Intraclass correlation coefficients (ICC) between ODIASP and the reference method after correction for systematic error, stratified by sex, BMI categories, and contrast enhancement.


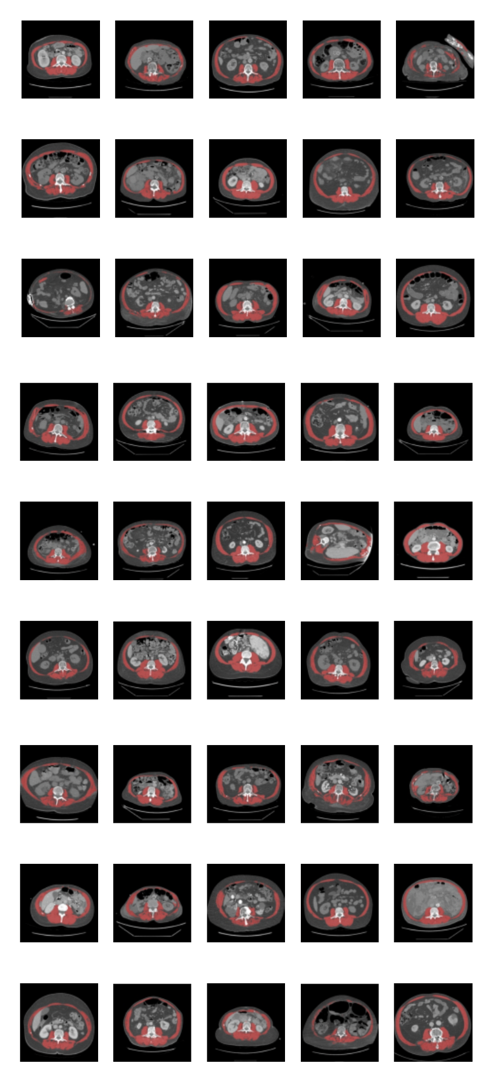


Supplementary Figure 2. Examples illustrating muscle segmentation at the L3 level on random CT scans processed by the ODIASP software.
